# Reducing Postoperative Opioid Consumption by Adding an Ultrasound-Guided Rectus Sheath Block to Multimodal Analgesia for Abdominal Cancer Surgery With Midline Incision

**DOI:** 10.5812/aapm.18263

**Published:** 2014-08-10

**Authors:** Ghada Mohammad Nabih Bashandy, Abeer Hassan Hamed Elkholy

**Affiliations:** 1 Department of Anesthesiology and Pain Management, National Cancer Institute, Cairo University, Cairo, Egypt

**Keywords:** Nerve Block, Postoperative, Ultrasound, Analgesia, Opioids, Multimodal

## Abstract

**Background:**

Many multimodal analgesia techniques have been tried to provide adequate analgesia for midline incisions extending above and below the umbilicus aiming at limiting the perioperative use of morphine thus limiting side effects. Ultrasound (US) guidance made the anesthesiologist reconsider old techniques for wider clinical use. The rectus sheath block (RSB) is a useful technique under-utilized in the adult population.

**Objectives:**

Our study examined the efficacy of a preemptive single-injection rectus sheath block in providing better early postoperative pain scores compared to general anesthesia alone.

**Patients and Methods:**

Sixty patients were recruited in this randomized controlled trial. These patients were divided into two groups: RSB group had an RSB after induction of anesthesia and before surgical incision, and GA (general anesthesia) group had general anesthesia alone. Both groups were compared for verbal analogue scale (VAS) score, opioid consumption and hemodynamic variables in the post-anesthesia care unit (PACU). Analgesic requirements in surgical wards were recorded in postoperative days (POD) 0, 1 and 2.

**Results:**

The median VAS score was significantly lower in RSB group compared with GA group in all 5 time points in the PACU (P ˂ 0.05). Also PACU morphine consumption was lower in RSB group than GA group patients (95% confidence interval [CI] of the difference in means between groups, −4.59 to −2.23 mg). Morphine consumption was also less in the first 2 postoperative days (POD0 and POD1).

**Conclusions:**

Ultrasound-guided rectus sheath block is an easy technique to learn. This technique, when it is used with general anesthesia, will be more effective in reducing pain scores and opioid consumption compared with general anesthesia alone.

## 1. Background

Abdominal surgeries requiring extended midline incisions are associated with severe postoperative pain. Many multimodal analgesia techniques have been tried to provide adequate analgesia for such incisions aiming at limiting the perioperative use of morphine thus limiting side effects (1). Recent randomized clinical trials (RCT) evidence showed that ultrasound guided regional techniques can offer an effective component of multimodal postoperative analgesia after a variety of surgeries with limited side-effects (2). Ultrasound (US) guidance is a safe and effective means to facilitate correct needle placement and adequate spread of local anesthetic for truncal blocks (3). It is important to remember that US does not lessen the anesthesiologist’s responsibility for using time-proven precautions (like repeated aspirations before incremental local anesthetic injections) for patient safety (4). However the extensive origin of the nerves that must be blocked (T5-T12 and L1) to provide analgesia for large midline abdominal incisions is considered a challenging issue. The mid or low thoracic and lumbar epidural analgesia has remained the ‘gold standard’ for providing good analgesia after abdominal surgery. Unfortunately, it is not always possible to provide epidural analgesia. The shift towards fast-track surgery protocols; the general unavailability of the monitored beds and risk of sepsis or coagulopathy may necessitate avoiding a central neuraxial block for analgesia.

Transversus abdominis plane (TAP) block has gained popularity in recent years (5). However, a satisfactory block for midline incisions extending above and below the umbilicus is not completely achieved by just TAP block approaches. It was proposed that the sensory block for posterior TAP block is T10 to L1. While subcostal TAP block is from T5 to T9. So these two blocks are complementary and if used in combination can provide analgesia from T5 to L1 segments. Of course, this procedure is considered impractical because large volumes of local anesthetic cannot be used safely (6, 7).

 The rectus sheath block (RSB) is an old technique that gained new clinical interest (8). It was first introduced into clinical practice in 1899 (9). By then it was used to achieve operative muscle relaxation and analgesia. RSB is assumed to suite midline incisions (10). Like other central non-neuraxial regional anesthesia, blocks of the abdomen RSB only provides analgesia for somatic pain, not pain of visceral origin (11). The anterior branches of the lower six thoracic and first lumbar sensory nerves travel in the TAP and enter medially into the rectus sheath, passing between rectus muscle and the posterior sheath. They penetrate anteriorly through rectus muscle ending by supplying the skin from the midline to approximately the anterior superior iliac spine (12). The local anesthetic (LA) can be deposited between the muscle and the posterior rectus sheath as anterior insertions of the arcuate lines limit the spread of LA solution anteriorly (13). Because, the lower abdomen nerves have a progressively shorter course, too medially deposited local anesthetic in the rectus sheath plane may miss all the nerves (14).

## 2. Objectives

Our current study examined the efficacy of US guided RSB to cover extensive midline abdominal incisions as a component of multimodal analgesia for midline laparotomies for radical cancer resections. Our primary end point was to examine whether a single pre-incision RSB could provide satisfactory early postoperative analgesia or not. The secondary end point was the possibility of such block to decrease postoperative morphine consumption and its side effects.

## 3. Patients and Methods

This prospective randomized observer-blinded controlled study was performed in National Cancer Institute of Cairo University "NCI-Cairo". The study was performed between February 2012 and December 2013. After approval of NCI-Cairo/IRB, written informed consents were obtained from all patients. Our study protocol recruited patients scheduled for elective surgeries for radical cancer resection needing extensive (extending from xiphisternum to symphysis pubis) midline incisions. Sixty patients with ASA (American Society of Anesthesiologists) physical status I-III, 18 to 75 years old, were randomly allocated to one of the following two groups with the intension to treat applied principles:

1. Combined general rectus sheath block anesthesia (group-RSB): where 20 mL of 0.25% bupivacaine in saline were injected into the rectus sheath plane on either sides under direct US visualization.
2. General anesthesia (group GA): where no RSB was performed.

Exclusion criteria were as follows: ASA physical status ≥ III; any contraindications to regional techniques (allergy to local anesthetics, infection around the site of the block, and coagulation disorder), history of analgesics dependence and any difficulty with communication.

The day before surgery, patients were instructed in the verbal analogue scale (VAS) score. The VAS scores with 0/10 representing no pain and 10/10 the worst imaginable pain. Patients were asked to score pain before operation on intravenous catheter insertion.

In the pre-surgical holding area, peripheral IV access was obtained, and all patients were pre-medicated with midazolam (3-5 mg IV) shortly before transfer to the operating room. Anesthesia was induced with fentanyl 2-3 µg/kg and propofol 2-2.5 mg/kg, IV route. An IV bolus of cisatracurium 0.1 mg/kg IV was given to facilitate tracheal intubation. After endotracheal intubation, patients were ventilated in a pressure-controlled volume guaranteed mode at tidal volumes less than 6 mL/kg, at respiratory rates to maintain end-tidal carbon dioxide concentration between 30–40 mmHg, with a positive end-expiratory pressure of 5 mmHg, and an inspired oxygen fraction (FIO2) of 0.6. Anesthesia was maintained with sevoflurane in oxygen and additional bolus doses of fentanyl 0.5–1 µg/kg to keep arterial pressure values around 20% below baseline values. Total intraoperative fentanyl consumption was recorded. After reversal of neuromuscular blocking agent and response to verbal command, patients were extubated in the operating theatre. They were then transferred to the PACU.

### 3.1. Rectus Sheath Block Technique

All rectus sheath blocks (RSB) were performed by one investigator in the operating room (OR) just after induction of anesthesia and before surgical incision. Basic intraoperative monitoring, according to ASA guidelines were used during the procedure and emergency equipment to respond to local anesthetic toxicity must be readily available. Complete aseptic technique was adopted.

**Figure 1.**
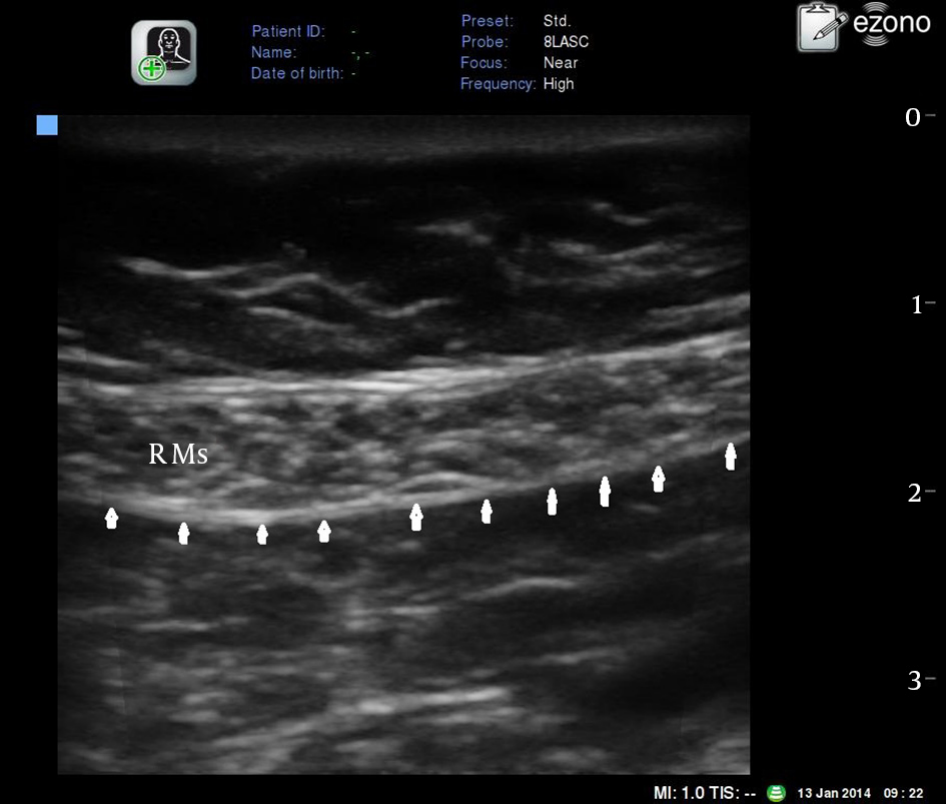
Transverse Ultrasound View of the Rectus Muscle Just Above the Umbilicus R Ms: rectus abdominis muscle. Arrows: posterior rectus sheath.

The rectus muscle is imaged with the ultrasound probe in a transverse orientation at or immediately above the level of the umbilicus (Figure 1). A broadband (5-12 MHz) linear array probe of eZono ™ 3000 ultrasound (eZono AG – Spitzweidenweg, Germany) was used, with an imaging depth of 4-6 cm.

Inserting the needle: An 18G Tuohy needle is introduced few millimeters from the probe using an in plane technique in an angle of approximately 45 degrees to the skin. The ultrasound image allowed identification of the rectus muscle and two hyperechoic railway-like lines deep in it (posterior rectus sheath and fascia transversalis) (Figure 1). The small size of the somatic sensory nerve fibers; located in this plane typically prevents their visualization by US or localization by nerve stimulation.

**Figure 2.**
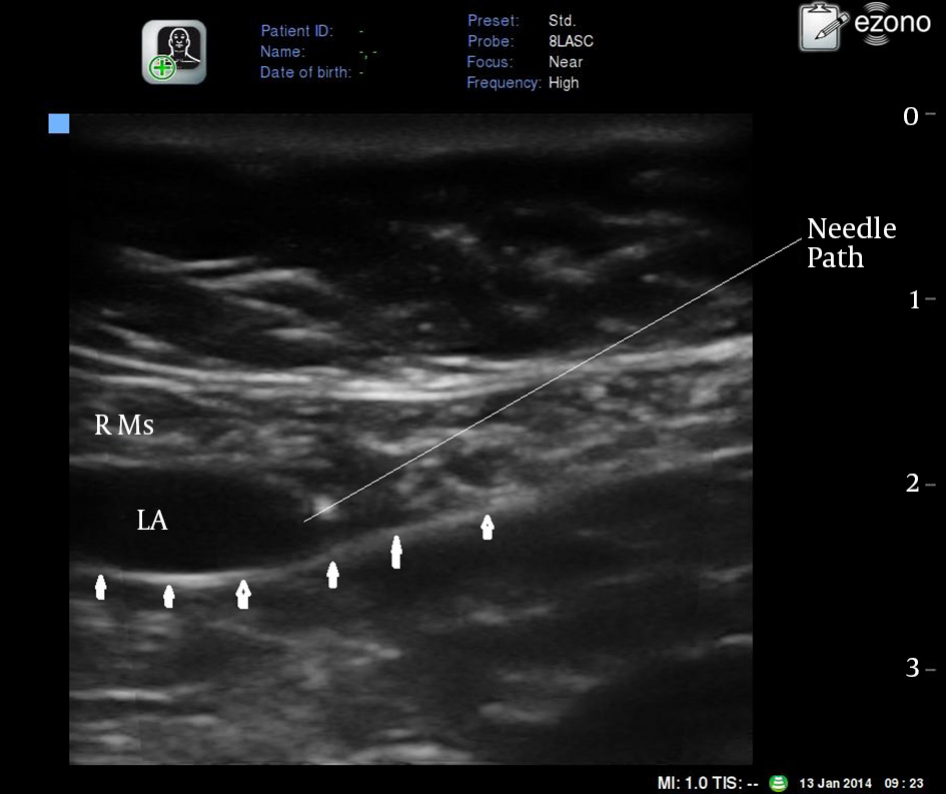
Transverse Ultrasound View of the Rectus Muscle Just Above the Umbilicus with Local Anesthetic Below the Rectus Muscle R Ms: rectus abdominis muscle. Arrows: posterior rectus sheath. LA: local anesthetic.

Under direct vision, the needle tip was advanced to the desired position where 20 mL bupivacaine 0.25% were injected causing hydrodissection of the rectus muscle away from the posterior rectus sheath (Figure 2). The technique is repeated on the opposite side.

In the PACU,

•All patients received IV infusions of paracetamol (Perfalgan) (15 mg/kg administered over 20 minutes then continued every 8 hours) and liometacen on request.
•Both groups had pain assessment when sufficiently awake for it, and IV morphine titration were performed by an attending anesthesiologist blinded to group allocation.
•Subsequently VAS pain scores were recorded every 15 minutes, in the PACU till discharge from it to the surgical ward. In the surgical ward VAS pain scores were assessed every 6 hours during the day of surgery (POD0) and next 2 days (POD1 and POD2).
•When the VAS score exceeded 3/10, IV Morphine 1-2 mg was administered and repeated at 5 minutes intervals until the VAS score decreased to < 3/10 at rest, and morphine consumption were recorded in the PACU, on POD0, POD1 and POD2.
•Respiratory rate, heart rate, and arterial pressure were recorded every 30 minutes, in the PACU and then every 2 hours after discharge to the ward. Respiratory depression was defined as a respiratory rate < 8 bpm.
•Patient sedation was assessed on a 5-point sedation Ramsay's score (1, wide awake; 2, drowsy or dozing intermittently; 3, mostly sleeping but easily awakened; 4, asleep, difficulty responding to verbal commands; 5, awakened only by shaking) (15) Oversedation was defined as having a sedation score > 4 combined with a respiratory rate < 8 bpm.
•Any incidence of nausea and vomiting PONV were also recorded where 0 = no nausea or vomiting, 1 = nausea, 2 = vomiting.

The primary outcome measures were pain intensities assessed by VAS score. Secondary outcome measures included consumption of IV morphine and incidence of respiratory depression, degree of sedation, nausea and vomiting.

### 3.2. Statistical Analyses

The data were expressed as mean ± standard deviation in normally distributed data. Statistical analysis was performed by independent t test for determining intergroup comparison and paired t test for intragroup difference. Data were expressed as median (interquartile range). Statistical analysis was performed using the Mann–Whitney U test for determining intergroup comparison. Chi-square test was used for categorical data. P ˂ 0.05 was considered statistically significant. SPSS 15.0 version was used for all the analysis.

## 4. Results

Sixty patients were enrolled in the study. Data from 56 patients, 29 from RSB group, and 27 from GA group were included in the final analysis. Two patients from GA group, who had consented to our protocol refused to participate in our study on the day of surgery. Two patients, one from GA group and one from RSB group was transferred to the intensive care unit for extensive intraoperative hemorrhage. Those patients were excluded from our study too. Seven patients, 2 from RSB group and 5 from GA group, were proved inoperable on surgical exploration because radical resection due to cancer metastases and/or local unresectablity. However, these patients were included in the final analysis on an intention-to-treat basis.

Demographic data for all 56 patients are shown in Table 1. Age, gender and ASA scores were not statistically different between the two study groups (P > 0.05). Types of surgery, length of surgery and estimated surgical blood loss (EBL) were also the same in the study groups (all P > 0.05). Baseline hemodynamic values "mean arterial blood pressure (MAP) and heart rate (HR)" were matched between RSB group and GA group (Table 1).

**Table 1.** Demographic and Intraoperative Data ^a,b^

Characters	RSB Group; (n = 29)	GA Group; (n = 27)	P Value
**Age, y**	49 ± 12	50.7 ± 8	0.550
**Gender**			
Male	13 (23.2)	15 (26.8)	0.422
Female	16 (28.6)	12 (21.4)	
**ASA I**	9 (16.1)	10 (17.9)	0.698 ^c^
**ASA II**	14 (25)	11 (19.6)	
**ASA III**	5 (10.7)	5 (8.9)	
**MAP0, mmHg**	82.4 ± 10	91 ± 10	0.131
**HR0, bpm**	83.7 ± 14.6	84.9 ± 14.2	0.766
**Surgical duration, min**	152.7 ± 79.9	166.6 ± 63.8	0.483
**EBL, mL**	800 (600-1000)	900 (500-1100)	0.540
**Type of Surgery**			0.809
Radical Cystectomy and Urinary Diversion	10 (17.9)	11 (19.6)	
Ovariectomy	8 (14.3)	6 (10.7)	
Hysterectomy	5 (8.9)	7 (12.5)	
Colectomy	4 (7.1)	2 (3.6)	
Anterior Pelvic Resection	2 (3.6)	1 (1.8)	

^a^ Abbreviations: ASA, American Society of Anesthesiologists; MAP 0, baseline mean arterial blood pressure; HR0, baseline heart rate; EBL, estimated surgical blood loss.

^b^ Data are expressed as median (interquartile range; Q1-Q3), mean ± standard deviation or number of patients No. (%).

^c^ Contingency coefficient test .

There was no statistically significant difference in supplementary fentanyl (223.3 ± 73.2 vs. 188.9 ± 65.4 µg) during operation to maintain HR and ABP 20% lower than the baseline values (P ˃ 0.05). The median VAS score was significantly lower in RSB group compared with GA group in all 5 time points in the PACU (P ˂ 0.05) (Table 2). PACU morphine consumption in RSB group was lower than GA group patients (95% confidence interval (CI) of the difference in means between groups, −4.59 to −2.23 mg). Morphine consumption mean in POD0 was statistically lower in RSB group (95% CI, − 6.65 to −4.78 mg). In POD1, morphine consumption in RSB group was also lower than GA Group (95% CI, −9.58 to −7.3 mg). However, both groups did not need any morphine in POD2 (Table 2).

**Table 2.** VAS Scores in the PACU and Postoperative Opioid Consumption ^a^

	RSB Group, (n = 29)	GA Group, (n = 27)	P value
**VAS1 (0 min)**	3 (3,5.5)	7 (6,9)	0.001 ^b^
**VAS2 (15 min)**	3 (2,3)	5 (3,6)	0.001 ^b^
**VAS3 (30min)**	2 (2,3)	3 (3,4)	0.01 ^b^
**VAS4 (45 min)**	2 (1.5,2)	3 (2,3)	0.015 ^b^
**VAS5 (60 min)**	2 (1,2)	3 (2,3)	0.02 ^b^
**PACU ^c^ Morphine consumption, mg**	2.1 ± 2.2	5.5 ± 2.1	0.001 ^b^
**POD0 ^c^ Morphine consumption, mg**	0.7 ± 1.3	6.4 ± 2	0.001 ^b^
**POD1 Morphine consumption, mg**	0	8.4 ± 3	0.001 ^b^

^a^ Data are presented as median (Q1, Q3) or mean ± SD.

^b^ statistically significant.

^c^ Abbreviations: PACU, post anesthesia care unit; POD, post anesthesia day.

**Figure 3.**
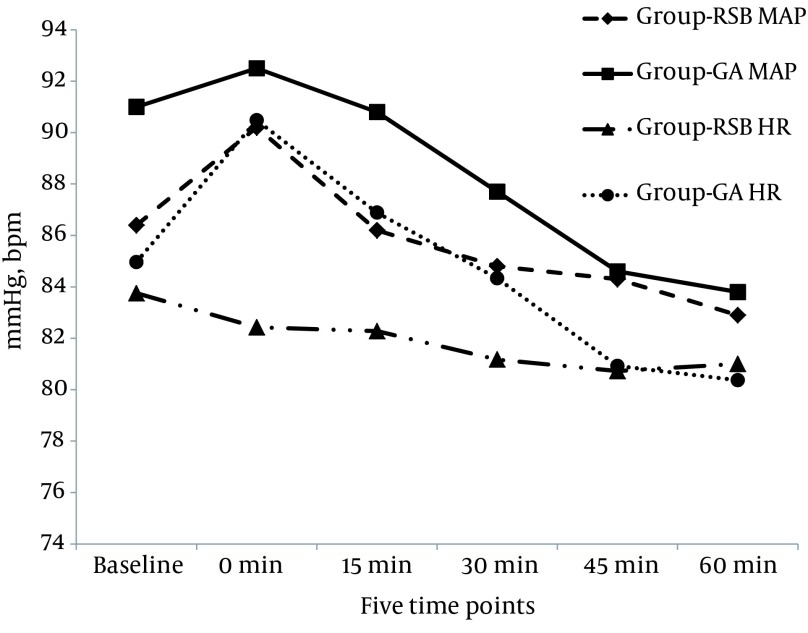
Hemodynamic Variability in the Two Groups in the PACU MAP: Mean arterial blood pressure. HR: Heart rate.

Median for incidence of postoperative nausea and vomiting in the PACU were less in RSB group than GA group [1 (1,1) vs. 1 (1,3) with P value = 0.027]. Mean respiratory rates of patients in GA group were lower than RSB group [11.8 ± 2.7 vs. 16.8 ± 4.2 with P value = 0.001]. However; no statistically significant difference in percentage of oxygen saturation of both groups in the PACU [99.3 ± 0.8 vs. 99.1 ± 1 with P value = 0.359]. Also median sedation scores was statistically significant higher in GA group than RSB group [3 (3,4) vs. 2 (1.5,3) with P value = 0.001]. Hemodynamic variables in the PACU recorded at the same time points as the VAS is depicted in Figure 3. The values for mean arterial blood pressure, heart rate, did not differ significantly between groups in each of the five time points (P ˃ 0.05). Heart rates in GA group were statistically higher than RSB group in the first time point (P ˂ 0.05). When we compared these values with the base line preoperative values; no statistically significant difference in HR or MAP were seen among RSB group patients. In GA group the MAP was higher than the baseline in the first time point MAP1 (91 ± 10.3 vs. 92.5 ± 8.9 mmHg, P = 0.005) and lower in last 2 time points MAP 4 and MAP 5 (91 ± 10.3 vs 84.6 ± 8 and 91 ± 10.3 vs. 83.8 ± 7.6 mmHg respectively) P = 0.001. Heart rate in the HR4 and HR5 time points was lower than the baseline (84.9 ± 14.2 vs. 80.9 ± 9.5 and 80.3 ± 9.2 bpm with P = 0.025 and 0.013 respectively) (Figure 3).

The procedure of rectus sheath block was uneventful; no side effects were observed in all cases like local hematoma or viscous puncture. There were also no signs of systemic local anesthetic toxicity.

## 5. Discussion

This study demonstrated that rectus sheath block with general anesthesia provides more effective pain relief than general anesthesia alone. Patients of RSB group had better VAS scores with less morphine utilization in the PACU and achieved more hemodynamic stability. They also consumed less opioid in the early postoperative period and consequently, developed fewer side effects like respiratory depression, excessive sedation, and postoperative nausea and vomiting.

The concept of multimodal analgesia has assumed increasing importance in the management of perioperative pain. It involves the use of different modalities of analgesia to provide superior pain relief with reduced individual analgesic-related side effects (16). Ultrasound-guided regional analgesia techniques are now widely accepted to supplement multimodal strategies (2).

The rectus sheath block is a useful technique under-utilized in the adult population. The large recognizable size of the rectus muscle made RSB an easy technique to master (3). RCT compared the performance of trainees using ultrasound versus loss-of-resistance (LOR) technique. Given the inexperience of trainees with both approaches, it was observed that the needle was placed in the correct tissue plane twice as often using ultrasound. In 21% of the LOR technique, the needle was placed intraperitoneal (17).

According to the previous studies, RSB has been used in adult population only in few small trials or case reports (10, 18-20). An early study has assessed the effect of intermittent injection of bupivacaine into rectus sheath space on postoperative opioid requirement, pain score and peak expiratory flow rate. Patients undergoing midline laparotomy received either bupivacaine 0.25% or normal saline via surgically placed catheters in the rectus sheath for 48 hours postoperative. No statistically significant differences in postoperative opioid requirement, pain score or PEFR were seen between two groups (21). Then a retrospective study recruiting 98 patients undergoing major gynecological surgery for benign or malignant disease received either standard subcutaneous infiltration of the wound with local anesthetic or the surgical RSB for post-operative pain relief. Patients who received the surgical rectus sheath block had lower pain scores on waking and required less morphine postoperatively compared to patients receiving standard subcutaneous local anesthetic into the wound (22).

Information regarding the pharmacokinetics of local anesthetics used in RSB is lacking. A recent study tested the detailed time course of ropivacaine concentrations after RSB. Thirty-nine patients undergoing elective lower abdominal surgery were randomized to 3 groups receiving RSB with 20 mL of different concentrations of ropivacaine. Peak plasma concentrations were dose-dependent, and there were no significant differences at the times to peak plasma concentrations. Furthermore; present data suggested slower absorption kinetics for ropivacaine after RSB than other compartment blocks (23).

One limitation of our study was that we evaluated only a single-injection RSB, a continuous infusion catheter or intermittent LA injections was not studied. However; we assumed that managing two catheters would be a bit difficult. Adding adjuvants to LA may improve efficacy and duration of RSB that can be tried in future studies. Studying the spread of local anesthetic and contrast solution into the Rectus sheath using magnetic resonance is recommended for future studies. This will help in appreciation of the pattern of spread of local anesthetic in the posterior rectus sheath in different sites of injection as well as single and multiple injections. This will have important implications for the extent of analgesia produced with each approach.

In conclusion, Ultrasound-guided rectus sheath block is an easy technique to learn. This technique, when used with general anesthesia, was found effective in reducing pain scores and opioid consumption compared to general anesthesia alone.
